# Inspiratory Muscle Training Based on Anaerobic Threshold on the Functional Capacity of Patients After Coronary Artery Bypass Grafting: Clinical Trial

**DOI:** 10.21470/1678-9741-2019-0448

**Published:** 2020

**Authors:** André Luiz Lisboa Cordeiro, Hayssa de Cássia Mascarenhas, Lucas Landerson, Jaclene da Silva Araújo, Daniel Lago Borges, Thiago Araújo de Melo, André Guimarães, Jefferson Petto

**Affiliations:** 1 Department of Human Medicine and Health, Escola Bahiana de Medicina e Saúde Pública, Unidade Acadêmica Brotas, Salvador, Bahia, Brazil.; 2 Department of Physiotherapy, Faculdade Nobre de Feira de Santana, Feira de Santana, Bahia, Brazil.; 3 Department of Physiotherapy, Cardiological Intensive Care Unit, Hospital Universitário da Universidade Federal do Maranhão, São Luis, Maranhão, Brazil.; 4 Department of Physiotherapy, Universidade Salvador, Salvador, Bahia, Brazil.; 5 Department of Cardiovascular Surgery, Instituto Nobre de Cardiologia, Feira de Santana, Bahia, Brazil.; 6 Department of Physiotherapy, Faculdade Adventista da Bahia, Cachoeira, Bahia, Brazil.; 7 Department of Physiotherapy, Faculdade Social da Bahia, Salvador, Bahia, Brazil.

**Keywords:** Muscular Strength, Myocardial Revascularization, Respiratory Muscles

## Abstract

**Introduction:**

Coronary artery bypass grafting (CABG) is associated with reduced ventilatory muscle strength and consequent worsening of functional capacity (FC). Inspiratory Muscle Training (IMT) can be indicated, but there is still a lack of knowledge about the use of the anaerobic threshold (AT) as a basis for prescription. The objective of this study is to evaluate if IMT based on AT modifies FC and inspiratory muscle strength of patients submitted to CABG.

**Methods:**

This is a clinical trial. On the first postoperative day, the patients were divided into two groups: the conventional group (IMT-C), which performed IMT based on 40% of maximal inspiratory pressure (MIP), and the IMT-AT group, which performed IMT based on AT. All patients underwent preoperative and postoperative assessment of MIP and performed a six-minute walk test (6MWT).

**Results:**

Forty-two patients were evaluated, 21 in each group. Their mean age was 61.4±10 years and 27 (64%) of them were male. There was a reduction of inspiratory muscle strength with a delta of 23±13 cmH2O in the IMT-C group *vs*. 11±10 cmH2O in the IMT-AT group (*P*<0.01) and of the walking distance with a delta of 94±34 meters in the IMT-C group *vs*. 57±30 meters in the IMT-AT group (*P*=0.04).

**Conclusion:**

IMT based on AT minimized the loss of FC and inspiratory muscle strength of patients submitted to CABG.

**Table:** 

Abbreviations, acronyms & symbols				
6MWT	= Six-minute walk test		IMT	= Inspiratory Muscle Training
AMI	= Acute myocardial infarction		IMT-AT	= Inspiratory Muscle Training based on anaerobic threshold
AT	= Anaerobic threshold		IMT-C	= Conventional Inspiratory Muscle Training
BMI	= Body mass index		MEP	= Maximal expiratory pressure
CABG	= Coronary artery bypass grafting		MIP	= Maximal inspiratory pressure
CI	= Confidence interval		MV	= Mechanical ventilation
DLP	= Dyslipidemia		PEF	= Peak expiratory flow
DM	= Diabetes mellitus		REP	= Repetitions
ECC	= Extracorporeal circulation		SAH	= Systemic arterial hypertension
FC	= Functional capacity		VC	= Vital capacity

## INTRODUCTION

Despite all technological advances, coronary artery bypass grafting (CABG) surgery is associated with ventilatory muscle dysfunction, which leads to an increase in the incidence of postoperative pulmonary complications and a decrease in functional capacity, ventilatory muscle strength, and pulmonary function^[1,2]^.

This decline in ventilatory muscle strength and pulmonary function has been well described in the literature by reducing maximal inspiratory pressure (MIP), maximal expiratory pressure (MEP), and vital capacity (VC). In addition, functional capacity can be measured by walking distance in the six-minute walk test (6MWT)^[1,3]^.

Inspiratory Muscle Training (IMT) is a tool to reduce the deleterious effects caused by cardiac surgery. By imposing a resistance in the inspiratory phase, it restores the integrity of the inspiratory muscles, also preventing hypotrophy and fatigue through pressure linear load devices^[4]^.

Several studies show that the conventional way to prescribe IMT is through a preestablished load according to the patient's MIP^[5,6]^. However, based on exercise physiology, we know that biological individuality and specificity are fundamental when we think of prescription and training to improve performance^[7]^.

In order to individualize the training, the anaerobic threshold (AT) can be used, which is represented by the variation of the glycemic curve^[7,8]^. Cordeiro et al.^[3]^ verified that the conventional IMT (based on 40% of the patient's MIP) resulted in less loss of inspiratory muscle strength and functional capacity. However, there is still a shortage in the literature of studies addressing the prescription of IMT based on AT of inspiratory muscles - if it yields similar or better results for reducing postoperative loss of functional capacity.

Therefore, our study aims to evaluate whether the IMT based on AT is superior to the conventional method regarding functional capacity and inspiratory muscle strength of patients undergoing CABG. A secondary objective is to assess the impact of IMT on pulmonary function and length of hospital stay.

## METHODS

This is a randomized controlled trial done with patients undergoing CABG at the Instituto Nobre de Cardiologia in Feira de Santana, Bahia, Brazil, from January to October 2018. This study is registered in the Brazilian Registry of Tests, or ReBEC, with the number RBR-8dqrdq.

### Study Population

The study included patients of both genders, with coronary artery disease, aged from 30 to 70 years, and submitted to CABG with extracorporeal circulation and median sternotomy. Exclusion criteria were the use of intra-aortic balloon, surgical reintervention, death, presence of valvopathies, previous pneumopathy, patients who did not understand the proposed techniques, had hemodynamic instability during the assessment, or IMT and physical limitation, such as amputation, which compromised the functional capacity assessment.

### Sample Size

To calculate the sample size, we performed a pilot study with 10 patients. Using a standard deviation of 63 meters in the 6MWT - based on the pilot of the final individual IMT group and 112 meters relative to the standard deviation of the conventional IMT group from the Cordeiro et al.^[3]^ study -, we used a difference of 30 meters that is related to the clinically relevant distance^[9]^. For a 5% alpha and aiming to achieve a power of 80%, 42 patients were necessary, 21 in each group.

### Ethical Aspects

Our study was submitted to and approved by the Ethics and Research Committee of Faculdade Nobre, in Feira Santana, Bahia, Brazil, obtaining the approval number 2,366,995. All participants signed a free and informed consent form.

### Study Protocol

Participants of the study were randomized by simple draw for the conventional IMT group (IMT-C) or the IMT group based on AT (IMT-AT). In this draw, there were two balls with a paper in each referring to the groups; then, a member of the team on duty choose one of the balls, being that the patient’s group of allocation. No researcher had influence on the procedures adopted by the team, being the patient managed based on the protocol of the institution, which consists of noninvasive ventilation, breathing exercises, kinesiotherapy, cycloergometry, and ambulation, conducts performed in the postoperative period. Pulmonary function, respiratory muscle strength, and functional capacity were evaluated one day before surgery, in patients already admitted to the hospital, and at hospital discharge.

### Both protocols started on the first day after the surgical procedure and lasted until hospital discharge.

Clinical and surgical characteristics, such as diabetes mellitus, systemic arterial hypertension, dyslipidemia, acute myocardial infarction, and sedentary lifestyle, were collected. All these comorbidities were known through the patient's chart, except for the physical inactivity, where the International Physical Activity Questionnaire, or IPAQ, was applied in the long format, which evaluates 27 questions related to physical activities performed in a normal week. The patient who did not perform any physical activity for at least ten continuous minutes during the week was considered to be a sedentary^[10]^. The physical activity variable was related to the week preceding the surgical procedure.

### Measurement of Respiratory Muscular Strength

Preoperative assessment of inspiratory muscle strength, MIP, was performed using an Indumed® (São Paulo, Brazil) analogue manovacuometer. During the evaluation, a maximal expiration until the residual volume was requested, and then a maximal and slow inspiration to the total lung capacity was required; this test was done using the unidirectional valve method, being possible a flow through a hole of one millimeter, aiming to exclude the action of the buccinator, and repeated for three times, being used the highest value reached, as long as this value was not the last. MEP was evaluated using the same apparatus and the patient was instructed to perform a maximal inspiration until he reached his total pulmonary capacity, the mask was placed, and after that a maximum expiration was requested until the residual capacity was reached. The test was repeated three times and it was considered the highest value result, as long as this value was not the last^[11]^. Both tests were performed with the patient seated, lower limbs resting on the ground.

### Measurement of Pulmonary Function

To assess VC, it was used the analogue ventilometer Ferraris Mark 8 Wright Respirometer (Louisville, Colorado, Unite States of America). The ventilometer was unlocked, cleared, and soon after the facial mask was placed on the face of the individual. The patient underwent deep inspiration until he/she reached his/her total pulmonary capacity, and soon after a slow and gradual expiration until reaching the residual volume. After this, the ventilometer was locked and the result observed and noted. The test was repeated three times, being considered the highest value result^[12]^.

Peak expiratory flow was evaluated using the peak flow of the Mini Wright^®^ brand. During the evaluation, the patient was seated, with his head in a neutral position and a nasal clip to prevent air from escaping through the nostrils. The patient took a deep breath, until total pulmonary capacity, followed by forced expiration with the mouth in the device. After three measurements, the highest value was chosen and there could be no difference > 40 liters between measurements^[12]^.

### Measurement of Functional Capacity

The 6MWT was used following the recommendations of the American Thoracic Society, or ATS, being conducted in a 30-meter, flat, and totally obstacle-free corridor. Prior to the test, patients had a rest period of at least 10 minutes. During this period, they were evaluated for contraindications, blood pressure data (through Premium aneroid sphygmomanometer and 3M Littmann stethoscope), pulse oximetry (Rossmax), dyspnea level (Borg scale), heart rate (assessed by palpation of the radial artery and counting over a period of one minute), and respiratory rate (evaluated by verifying the respiratory incursion during one minute). The patient was advised to walk as fast as possible, without running, in this corridor for six minutes. During the test, encouragement phrases were used each minute. At the end of the test, the examiner quantified the distance covered within those six minutes^[12,13]^.

During the protocol, patients were monitored, and in the presence of an increase in systolic and/or diastolic blood pressure > 30% of baseline, heart rate < 20% of baseline, peripheral oxygen saturation < 90%, and increased respiratory rate > 25 breaths per minute, the test was discontinued^[12]^.

### Assessment of Length of Hospital Stay

The total length of hospital stay was established in days from admission to discharge.

### Inspiratory Muscle Training Protocol

IMT-C - Patients underwent MIP assessment and initiated IMT with a linear pressure loading device (PowerBreathe Knectic Series®, HaB International, United Kingdom), with 40% of MIP, performing three sets with 15 repetitions. This training was performed twice daily until the day of hospital discharge.

**Fig. 1:**
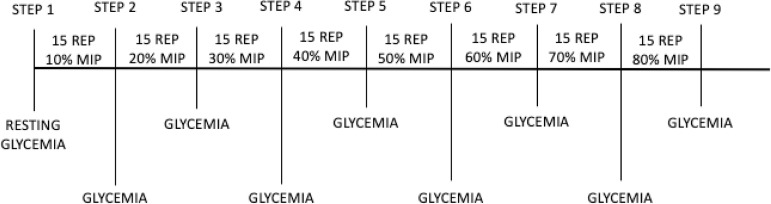
Glycemic threshold assessment protocol. MIP=maximal inspiratory pressure; REP=repetitions

IMT-AT - Patients in this group were submitted to exercise prescription according to the glycemic threshold on the first day after the surgical procedure. Resistance of the inhaling muscles was assessed by a maximal progressive test conducted at PowerBreathe Knectic Series® device (HaB International, United Kingdom). This test of inspiratory muscles, of non-continuous incremental characteristic, is composed of up to 10 stages of 15 repetitions with increasing increment of load. After the end of each step, a twominute interval was allowed. Using the same evaluation equipment, it started with 10% of the MIP value and increased 10% at each level of the test, and at the end of each load level, the capillary blood glucose was evaluated through the Accu-Chek Performa® device (Roche). The test was discontinued when the individual was no longer able to overcome the burden imposed by the device or expressed that he was unable to continue the test^[14]^. The load used for IMT corresponded to the lower glycemic value found between the loads. Therefore, a load of 10% of MIP was adjusted and after the established repetitions, the blood glycemia evaluation was performed; after the load adjustment to 20%, new repetitions and collection of blood glucose; after 30% of MIP, 15 repetitions and new blood glucose evaluation; this process occurred until the patient could not open the device valve or there was inability to continue. This evaluation protocol is illustrated in Figure 1. Measurement of the load was repeated every four days. The training was performed in three sets with 15 repetitions, twice a day until the day of hospital discharge.

### Statistical Analysis

The IBM SPSS Statistics software, version 20.0, was used for analysis. To verify normality, the Shapiro-Wilk test was used. Quantitative variables were expressed as mean and standard deviation. Categorical variables were analyzed using the Chisquare test. To compare the preoperative value with the value of intragroup hospital discharge, the paired Student's *t*-test or Mann-Whitney U test was used. To evaluate the values in the comparison between the groups, the independent Student’s *t*-test or Wilcoxon test was used. A *P*<0.05 was considered significant.

## RESULTS

**Fig. 2:**
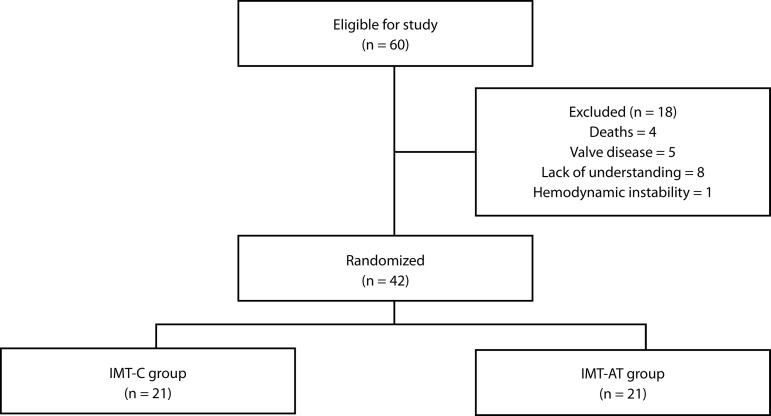
Study flowchart. IMT-AT=Inspiratory Muscle Training based on anaerobic threshold; IMT-C=conventional Inspiratory Muscle Training

During the study period, 60 patients were admitted for myocardial revascularization surgery, of which 42 completed the study (Figure 2). The sample characterization is shown in Table 1.

**Table 1:** Clinical and surgical data of patients undergoing coronary artery bypass grafting.

Variables	IMT-C group (n = 21)	IMT-AT group (n = 21)	*P*-value
**Gender**			
Male	13 (62%)	14 (67%)	0.45^a^
Female	8 (38%)	7 (23%)	
Age (years)	62±10	61±9.6	0.90^b^
BMI (kg/m^2^)	27±3.9	27±3.2	0.76^b^
**Comorbidities**			
DM	13 (62%)	9 (43%)	0.33^a^
SAH	11 (52%)	13 (62%)	0.41^a^
DLP	12 (57%)	8 (38%)	0.33^a^
Sedentary	13 (62%)	11 (52%)	0.10^a^
AMI	8 (38%)	7 (33%)	0.44^a^
MV time (hours)	7.6±2,1	8.1±2.1	0.31^b^
ECC time (min)	85.7±13.8	88.8±17.1	0.43^b^
Number of grafts	2.6±0.8	2.5±0.6	0.57^b^

a Chi-square test;

b Independent Student’s t-test

AMI=acute myocardial infarction; BMI=body mass index; DLP=dyslipidemia; DM=diabetes mellitus; ECC=extracorporeal circulation; IMT-AT=Inspiratory Muscle Training based on anaerobic threshold; IMT-C=conventional Inspiratory Muscle Training; MV=mechanical ventilation; SAH=systemic arterial hypertension

**Fig. 3:**
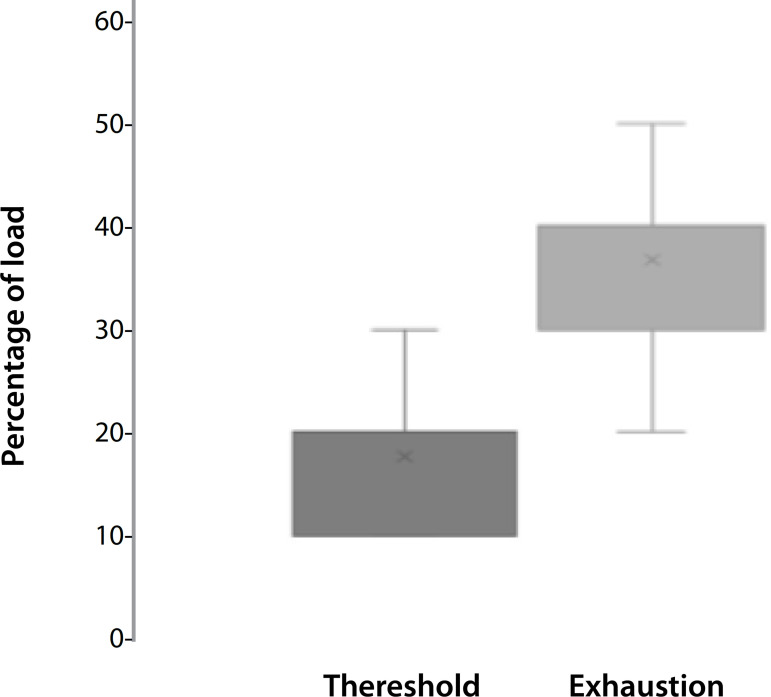
Threshold and exhaustion values of patients undergoing muscle training based on glycemic threshold.

Figure 3 shows the verified values of the glycemic threshold and the percentage of load when patients reached exhaustion. On average, patients presented a threshold of 20% of MIP, and exhaustion was achieved on average with 35% of the load. The load used for IMT in the IMT-AT group was lower than the load used by the IMT-C group.

In both groups, there was a reduction of VC and peak expiratory flow at hospital discharge when compared to the preoperative period. The inspiratory muscle strength showed a 32% reduction in the IMT-C group; in the IMT-AT group, the decrease was 12% when compared to the preoperative period and hospital discharge. Expiratory muscle strength also showed worsening in both groups, 27% in the conventional group and 26% in the individualized group (Table 2).

**Table 2:** Pulmonary function and ventilatory muscular strength, preoperatively and at hospital discharge, in conventional and individualized muscle training groups.

Variables	IMT-AT group (n = 21)	IMT-C group (n = 21)	CI (95%)	*P*-value
**MIP (cmH_2_O)**				
Preoperative	103±19	101±15	2 (-4 to 1)	0.78^a^
Hospital discharge	92±15^†^	77±14^†^	15 (9 to 19)	< 0.01^a^
Δ	11±10	23±13		< 0.01^a^
**MEP (cmH_2_O)**				
Preoperative	85±17	77±16	8 (-4 to 12)	0,34^a^
Hospital discharge	68±16^†^	61±11^†^	7 (-3 to 13)	0,33^a^
Δ	17±15	16±13		0,87^a^
**VC (ml/kg)**				
Preoperative	51±9	53±5	-2 (-5 to 4)	0.55^a^
Hospital discharge	44±6^†^	46±5^†^	-2 (-4 to 4)	0.44^a^
Δ	7±4	7±2		0.98^a^
**PEF (L/min)**				
Preoperative	420±137	384±131	-36 (-45 to 15)	0.41^b^
Hospital discharge	347±106^†^	312±108^†^	-35 (-41 to 9)	0.33^b^
Δ	73±56	71±55		0.93^b^

a Independent Student’s t-test;

b Mann-Whitney U test;

† *P*<0.01 of preoperative and discharge periods through the paired Student’s *t*-test

CI=confidence interval; IMT-AT=Inspiratory Muscle Training based on anaerobic threshold; IMT-C=conventional Inspiratory Muscle Training; MEP=maximal expiratory pressure; MIP=maximal inspiratory pressure; PEF=peak expiratory flow; VC=vital capacity

Δ = is the difference between the preoperative value and the discharge

In the evaluation of submaximal functional capacity, there was a worsening in both groups in the distance covered when comparing the preoperative period with hospital discharge. The IMT-C group showed a fall of 94±84 meters, while the IMTAT group showed a reduction of 57±30 meters (*P*=0.04). When comparing the groups at the time of hospital discharge, patients who performed individualized muscle training walked 37 meters longer than those in the conventional group (*P*<0.001). In percentage, the IMT-C group showed a 22% reduction, while the IMT-AT group had a 13% loss. Table 3 shows the information related to functional capacity. Table 3 also shows a reduction in the length of hospital stay of patients who received individualized muscle training.

**Table 3:** Capacity and functional independence, preoperatively and at hospital discharge, in conventional and individualized muscle training groups.

Variable	IMT-AT group (n = 21)	IMT-C group (n = 21)	CI (95%)	*P*-value^a^
**6MWT**				
Preoperative	429±71	426±75	3 (-5 to 9)	0.89^a^
Hospital discharge	373±55^†^	332±84^†^	41 (33 to 56)	< 0.01^a^
Δ	57±30	94±84		0.04^a^
**Length of stay (days)**	7±1.3	8.2±1.3	-1.2 (-2 to -0.1)	< 0.01^a^

a Independent Student’s t-test;

† *P*<0.01 of preoperative and discharge periods through the paired Student’s *t*-test

6MWT=six-minute walk test; CI=confidence interval; IMT-AT=Inspiratory Muscle Training based on anaerobic threshold; IMT-C=conventional Inspiratory Muscle Training

Δ= is the difference between the preoperative value and the discharge

## DISCUSSION

Based on the results, we found that IMT based on AT was effective in decreasing the loss of inspiratory muscle strength, improving the submaximal functional capacity assessed through the 6MWT. The possible reason for the relationship between muscle strength and performance in the walking test is the reduction of the metaboreflex induced by the optimization of the diaphragmatic function. So, there was an improvement in peripheral blood flow and increased functional capacity.

An important finding in patients after cardiac surgery is the reduction of ventilatory muscle strength, which can negatively affect quality of life, postoperative complications, and functional capacity^[6,15]^. Studies show that this decrease can reach 40% when comparing the preoperative period with the value of hospital discharge^[3,16]^. In the present study, there was a reduction of MIP in both groups, but it was milder in the group that performed training based on AT (a 12% reduction).

The reduction of inspiratory muscle strength is an expected finding after cardiac surgeries and physiotherapy has the role of minimizing the postoperative loss. These results become even more interesting when we consider that the mean percentage of exercise prescription was 20% of the strength, while in the conventional group 40% of the MIP was used. That is, the training was performed with an ideal load level for the diaphragm, having this muscle strength and endurance characteristics.

The diaphragm is a muscle with a predominance of type I fibers, followed by type IIa fibers and, to a lesser extent, type II b fibers, which have strength characteristics. Performing training with lower loads probably optimizes the type I and IIa fibers, which improves diaphragmatic performance, generating greater strength gain^[17]^.

In the study by Miozzo et al.^[18]^, they evaluated the repercussion of aerobic exercise associated with IMT in patients submitted to CABG. In that study, the training was started with a load corresponding to 50% of MIP and it was evaluated every 12 sessions, totaling 36. The authors did not verify significant impact of the application of IMT associated with aerobic exercise in the studied population. The difference between the result of Miozzo et al.^[18]^ and the present study may lie in the level of applied load. Probably, 50% of the maximum force causes the diaphragm to work above its resistance force characteristic, and it does not prove to be superior to the protocol applied by our group.

In this sense, the IMT-AT group performed better on the 6MWT at discharge when compared to the conventional group. Studies show that in cardiac patients the redistribution of blood flow during the metaboreflex to inspiratory muscles generates a reduction of the concentration of blood in peripheral muscles leading to a reduction of functional capacity^[19,20]^. With the IMT, there was a decrease of the metaboreflex in order to contribute to the better physical performance of this population.

During exercise, there are reflex cardiovascular changes (increased blood pressure and peripheral vascular resistance) that are induced by muscle metaboreflex, and these changes are linked to the development of acidosis during exercise, which in turn leads to worsening muscle performance^[21]^.

The difference in the walking test averaged 40 meters, which is not only statistically significant but also clinically relevant. In the study by Shoemaker et al.^[9]^, it was found that in patients with chronic heart failure the clinically significant distance is 30 meters. Gremeaux et al.^[21]^ show that the minimum significant difference is 25 meters in patients with coronary artery disease.

In the study by Stein et al.^[22]^, a positive correlation was found between MIP and peak oxygen consumption, making muscle shape a predictor of functional capacity in patients after CABG. Based on the results, we can infer that there was a reduction of muscle oxygen consumption, which may have contributed to an improvement in functional capacity in our study.

Increased functional capacity is associated with improved quality of life. Savci et al.^[24]^ verified that a 21% increase in the walking distance during the submaximal test was related to an increase in the quality of life of patients who performed IMT after CABG.

Bohannon and Crouch^[24]^, in a systematic review, concluded that a difference of 14 to 30.5 meters is associated with improved quality of life in groups of patients with different health conditions. A similar finding was verified by Wyrwich et al.^[25]^ in a study involving individuals with chronic and cardiac lung diseases.

Based on the abovementioned information and the results obtained in the present study, it can be inferred that a smaller decrease or decline on inspiratory muscle strength and functional capacity in patients undergoing IMT after cardiac surgery may be associated with improved quality of life at hospital discharge. An increase may be related to greater participation in society, favoring more productive and less restricted patients after hospital stay.

The length of hospital stay in the present study was, on average, one day shorter in the group that performed training based on glycemic threshold. This result has already been evidenced in other studies^[3,26,27]^. What is noteworthy once again is that the individualized group spent less time in hospital, used a lower load than the conventional group, and yet presented only a 12% reduction of MIP, while the conventional group showed a 32% decrease.

### Limitations

A limitation in the present study is the lack of application of an instrument for postoperative pain assessment, although analgesia was optimized for all patients. And an important limitation was the calculation based on patients with heart failure, since we do not have data available in the literature on the clinically important difference in patients undergoing cardiac surgery, which may reduce the power of the results.

## CONCLUSION

In conclusion, the IMT protocol based on AT minimized the loss of functional capacity, inspiratory muscle strength, and reduced the length of hospital stay of patients undergoing CABG.

**Table:** 

**Authors' roles & responsibilities**	
ALLC	Substantial contributions to the conception or design of the work; or the acquisition, analysis, or interpretation of data for the work; drafting the work or revising it critically for important intellectual content; agreement to be accountable for all aspects of the work in ensuring that questions related to the accuracy or integrity of any part of the work are appropriately investigated and resolved; final approval of the version to be published Substantial contributions to the conception or design of the work; or the acquisition, analysis, or interpretation of data for the work; final approval of the version to be published
HCM	Substantial contributions to the conception or design of the work; or the acquisition, analysis, or interpretation of data for the work; final approval of the version to be published
LL	Substantial contributions to the conception or design of the work; or the acquisition, analysis, or interpretation of data for the work; final approval of the version to be published
JSA	Final approval of the version to be published
DLB	Final approval of the version to be published
TAM	Final approval of the version to be published
AG	Substantial contributions to the conception or design of the work; drafting the work or revising it critically for important intellectual content;
JP	Final approval of the version to be published.
